# Multicolor Emission from Ultraviolet GaN-Based Photonic Quasicrystal Nanopyramid Structure with Semipolar In_*x*_Ga_1−*x*_N/GaN Multiple Quantum Wells

**DOI:** 10.1186/s11671-021-03576-1

**Published:** 2021-09-16

**Authors:** Cheng-Chang Chen, Hsiang-Ting Lin, Shih-Pang Chang, Hao-Chung Kuo, Hsiao-Wen Hung, Kuo-Hsiang Chien, Yu-Choung Chang, M. H. Shih

**Affiliations:** 1 Energy and Environment Research Laboratories, Industrial Technology Research Institute, Hsinchu, 31040 Taiwan; 2 Research Center for Applied Sciences (RCAS), Academia Sinica, Taipei, 11529 Taiwan; 3 Department of Photonics and Institute of Electro-Optical Engineering, National Chiao Tung University, Hsinchu, 30010 Taiwan

**Keywords:** GaN, GaN-based LEDs, Photonic quasicrystal multicolor emission, Finite-element method

## Abstract

In this study, we demonstrated large-area high-quality multi-color emission from the 12-fold symmetric GaN photonic quasicrystal nanorod device which was fabricated using the nanoimprint lithography technology and multiple quantum wells regrowth procedure. High-efficiency blue and green color emission wavelengths of 460 and 520 nm from the regrown In_*x*_Ga_1−*x*_N/GaN multiple quantum wells were observed under optical pumping conditions. To confirm the strong coupling between the quantum well emissions and the photonic crystal band-edge resonant modes, the finite-element method was applied to perform a simulation of the 12-fold symmetry photonic quasicrystal lattices.

## Background

The GaN-based materials with the wide band gap and unique properties had been applied in many optoelectronic systems and devices, including light emitting diodes (LEDs) [1–3] and laser diodes (LDs) [4, 5]. The GaN-based LEDs have been applied in traffic signals, display backlights [6–8], solid-state lighting [9, 10], biosensors [11], and optogenetics [12]. One of the challenges for the advanced GaN LEDs is to realize the phosphor-free white LEDs, including multichip white LEDs, monolithic LEDs, and color-conversion white LEDs [13, 14]. GaN-based nanorod LED with low dislocation, low internal field, and high light extraction efficiency [15, 16] could be a possible solution. Various approaches have been employed to increase the light extraction efficiency for III-nitride LEDs, such as rough surfaces [17–20], sapphire microlenses [21], oblique mesa structure [22], nanopyramids [23], graded refractive index materials [24], self-assembled lithography patterning [25], colloidal-based microlens arrays [26, 27], and photonic crystals [28–31]. Photonic crystals have been reported in quasicrystal or defective two-dimensional (2D) grating configurations and lead to improved light extraction efficiency in LEDs [32–35]. The photonic crystal structure is periodic with translational symmetry. The periodic structure can exhibit a photonic band gap to inhibit the propagation of guided modes and uses a photonic crystal structure to couple guided modes with radiative modes [36–39]. Photonic crystal lasers based on the band-edge effect have several advantages, such as high-power emissions, single mode operation, and coherent oscillation [40–42]. E-beam lithography and laser interference lithography have been used to produce the photonic crystal structure [43, 44]. Furthermore, because the emitting units are separated and the emission surfaces face each other, the light can be mixed effectively. Thus, nanorods are considered to have a great advantage for improving the luminous efficiency in the green-to-red emission region, and numerous efforts have been adopted [45, 46].

**Fig. 1:**
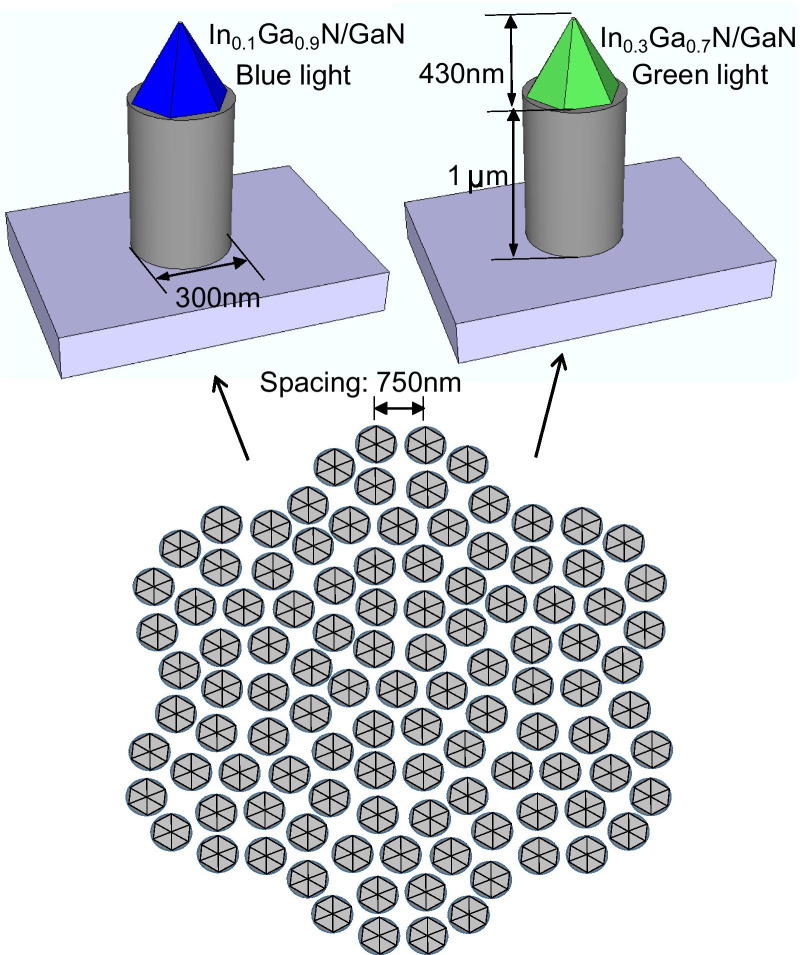
Schematic structure of GaN-based PQC structure with the regrowth of semipolar {10-11} GaN pyramids and 10-pair In_*x*_Ga_1−*x*_N/GaN (3 nm/12 nm) MQW

However, nanoimprint lithography (NIL) offers high-level resolution, low-cost, and high throughput compared with other forms of lithography including laser interference and e-beam lithography [47–49]. In this study, we demonstrated the multiple color emission from a GaN-based 2D photonic quasicrystal (PQC) structure as illustrated in Fig. 1. The PQC structure was fabricated using NIL [41, 42]. The total area of the PQC pattern is approximately 4 cm × 4 cm(2-in. sapphire substrate) and possessed 12-fold symmetry [50, 51], with a lattice constant of approximately 750 nm, a diameter of 300 nm, and the depth of the nanopillars is approximately 1 μm. The PQC structure formed a complete band gap with the regrowth of 430-nm-tall GaN pyramids and 10-pair semipolar {10-11} In_*x*_Ga_1−*x*_N/GaN (3 nm/12 nm) multiple quantum well (MQW) nanostructures, as illustrated in Fig. 1.

Under room temperature pumping operation, the device demonstrates laser action with a low threshold power density and the multiple color emission simultaneously. We had reported the single-color laser action from the GaN PQC structure [41, 42]. This PQC platform exhibits the advantages in low fabrication costs, and better integration of GaN-based material with multi-color systems. In the future, the multiple-color GaN-based lasers can be expected with the optimization of regrowth procedure and the high-quality photonic crystal cavity.

## Methods

### Design and Fabrication of Sample

**Fig. 2:**
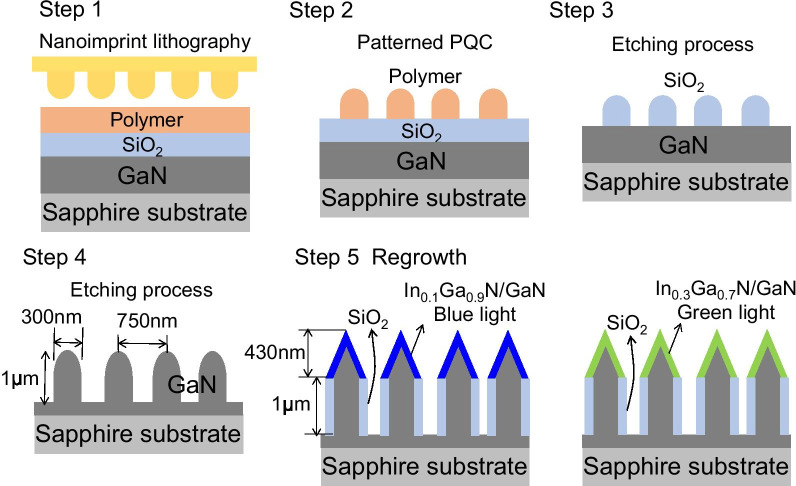
The schematic of the GaN PQC structure fabrication process. Including epitaxial growth of a GaN wafer (step 1), NIL of PQC patterns (step 2), dry etching (steps 3 and 4), and pyramid-on-nanorods MQW structure after regrowth (step 5)

Figure 2 illustrates the schematic procedures of the device fabrication. The fabrication procedures included epitaxial growth of a GaN wafer, NIL of PQC patterns, and dry etching. The GaN-based material was grown in a low-pressure metalorganic chemical vapor deposition reactor on a C-plane (0001) sapphire substrate. To prepare a clean surface of the sapphire substrate, the substrate was immersed into a burning solution of sulfuric acid: phosphoric acid = 3:1, then heat the beaker to a constant temperature for 1 h. The substrate was cleaned with DI water under ultrasonic oscillation. A GaN (1-μm thick) was first grown on a 2-inch sapphire substrate at 1160 °C. A 0.4-μm SiO_2_ mask and 0.2-μm polymer mask were then deposited. After the polymer film was dry, a patterned mold of a 2-inch PQC structure was placed onto it by applying high pressure (Fig. 2. step 1). The substrate was heated to higher than the polymer’s glass transition temperature (*T*_g_). The substrate and the mold were then cooled to room temperature to release the mold. The PQC patterns were defined on the polymer layer (Fig. 2, step 2). The patterns were then transferred into a SiO_2_ layer with reactive ion etching (RIE) by using a CHF_3_/O_2_ mixture (Figure, step 3). The SiO_2_ layer was used as a hard mask. The structure was then etched using inductively coupled plasma RIE with a Cl_2_/Ar mixture. The mask of SiO_2_ layer was removed at the end of the etching process (Fig. 2, step 4).

**Fig. 3:**
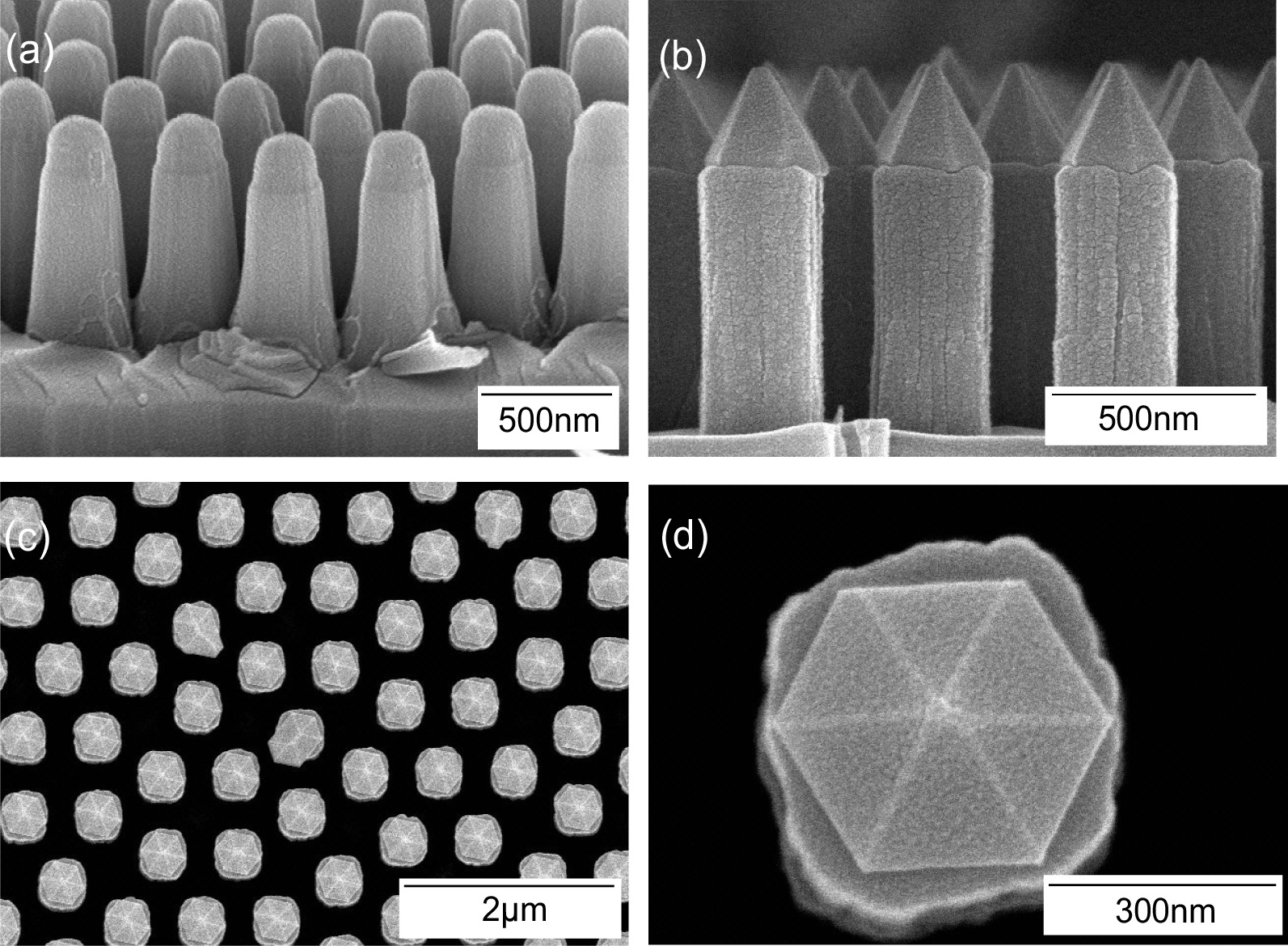
**a** The tiled angle-view SEM image of the PQC structure. **b** The cross-sectional-view SEM image of the PQC structure with porous SiO_2_. **c** Top-view SEM image of the PQC structure after the regrowth procedure. **d** Magnifying SEM image of semipolar {10-11} In_*x*_Ga_1−*x*_N/GaN MQW with the facets of trapezoid microstructures

Before the regrowth process, the sample was passivated with porous SiO_2_ at the sidewall of the nanopillars. The pyramid-shaped GaN structures were regrown on top of the GaN nanopillars at 730 °C. The 0.43-μm-high pyramids contained 10-pair In_x_Ga_1-x_ N/GaN (3 nm/12 nm) quantum wells, which supported different wavelengths of blue and green color emission, with the ratio of in composition: In_*x*_Ga_1−*x*_N/GaN-dependent InN fraction variations. In_0.1_Ga_0.9_N/GaN MQWs and In_0.3_Ga_0.7_N/GaN MQWs corresponded to 460- and 520-nm emission wavelengths, respectively (Fig. 2, step 5). The etch depth of the nanorods was approximately 1 μm, as illustrated in Fig. 3a. Figure 3b, c shows the SEM images of the PQC structure with the porous SiO_2_ layer and a semipolar {10-11} In_*x*_Ga_1−*x*_ N/GaN MQW. Figure 3d displays the magnification of semipolar {10-11} In_*x*_Ga_1−*x*_ N/GaN MQW with the facets of trapezoid microstructures. The semipolar {10-11} planes can reduce the influence of the quantum-confined Stark effect on the quantum efficiency of LEDs due to the surface stability and suppression of polarization effects [52–55].

To study the optical properties of the GaN-based PQC with nanopyramid structure, two GaN PQC samples were prepared: A, In_0.1_Ga_0.9_N/GaN MQWs, and B, In_0.3_Ga_0.7_N/GaN MQWs with regrowth fabrication. During the regrowth step, the temperature is the key to control the ratio of indium composition. The control temperature of blue In_0.1_Ga_0.9_N is 760–780 °C, and the control temperature of green In_0.3_Ga_0.7_N is 730–740 °C.

## Results and Discussion

**Fig. 4:**
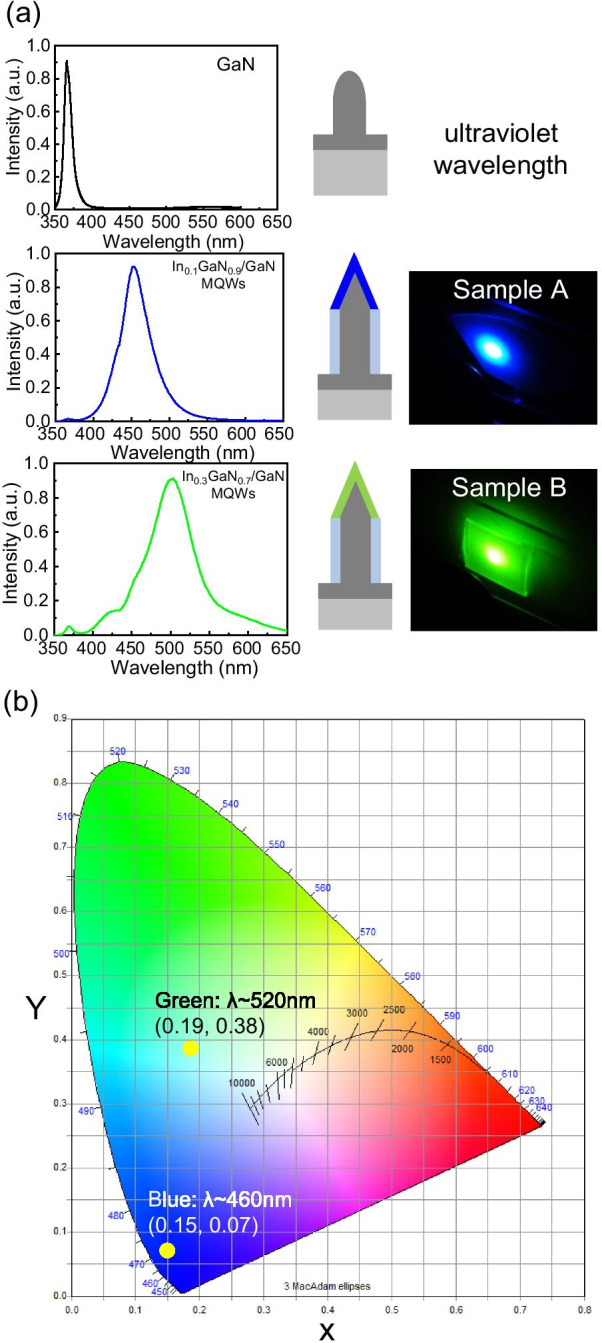
**a** PL spectra from the nanorods of GaN-based material (black), samples A (blue) and B (green). **b** Photographs of the PQC structure of samples A and B during measurement corresponding to the CIE coordinates of (0.19, 0.38) and (0.15, 0.07), respectively

To demonstrate the optical mode from the photonic quasicrystal structure, samples A and B were optically pumped by a continuous-wave (CW) He-Cd laser at 325 nm with an incident power of approximately 50 mW. The light emission from the device was collected by a 15 × objective lens through a multimode fiber, and coupled into a spectrometer with charge-coupled device detectors. Figure 4a illustrates the measured PL spectra under He-Cd 325 nm CW laser pumping. The spectrum of the black curve is the light emission with a wavelength of 366 nm from the GaN-based PQC structure displayed in Fig. 3a. Both samples A (blue curve) and B (green curve) had a strong emission peak which corresponded to wavelengths of approximately 460 and 520 nm, respectively, resulting from the In_x_Ga_1-x_ N/GaN MQWs structure. The spectrum linewidths of the samples A and B were 40 and 60 nm, respectively. Figure 4a also displays photographs of the PQC structure of samples A and B during measurement. The CIE coordinates of PL from samples A and B were (0.19, 0.38) and (0.15, 0.07), respectively, as illustrated in Fig. 4b. Thus, this hybrid platform has several possibilities for multicolor LEDs. It should be note that the peak of the sample B is broader than the one of sample A in Fig. 4a. The slight broad spectrum from the sample B was attributed to the existence of defects and dislocations generated by the higher indium composition [56–58].

**Fig. 5:**
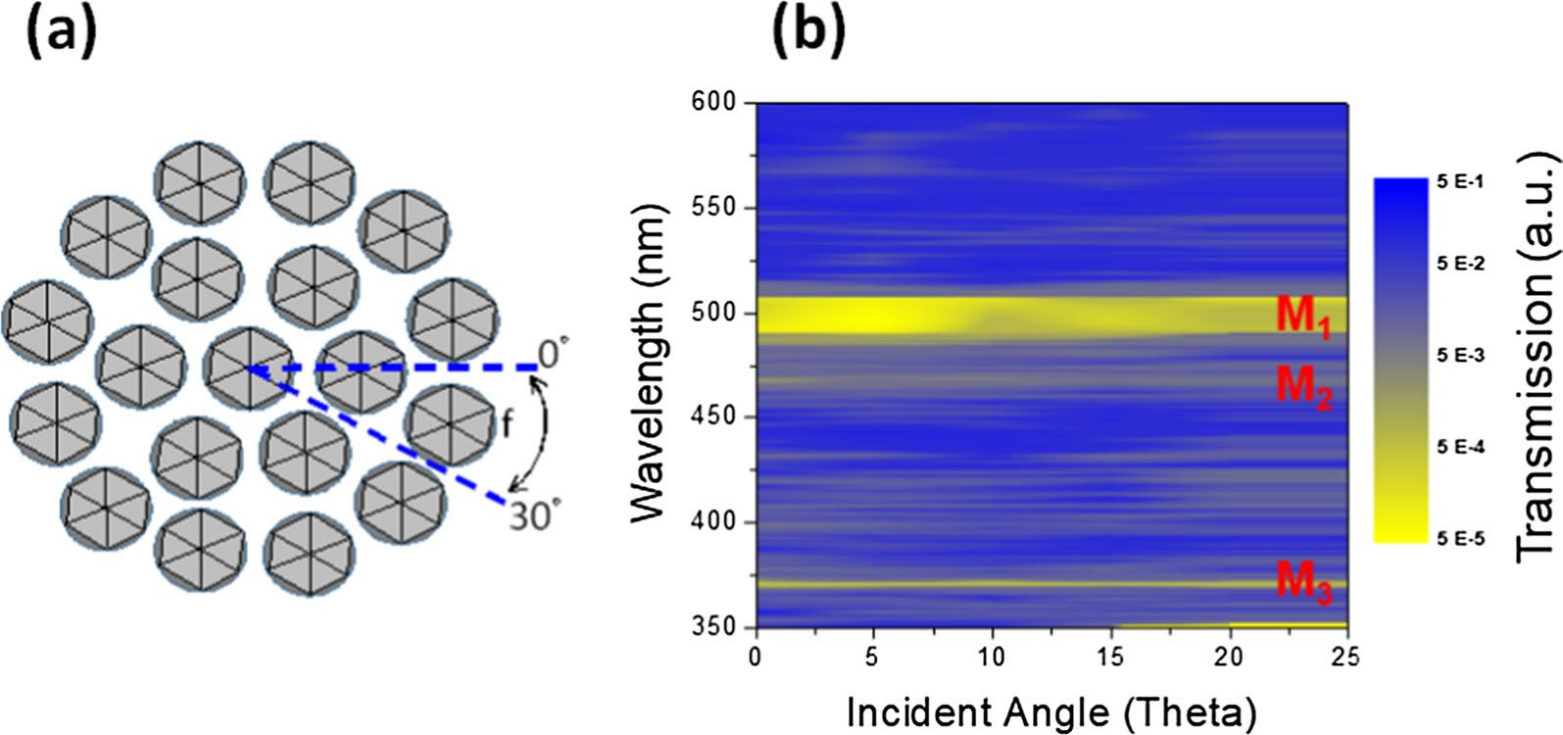
**a** Duplicate spectra for every 30° incident angle owing to the symmetry of the PQC structure. **b** Transmission spectrum of the 12-fold symmetry photonic quasicrystal lattices, calculated by FEM corresponding to different band-edge resonant modes

In order to confirm the optical resonant modes were the PQC band-edge modes, the finite-element method (FEM) [59, 60] was used to perform a simulation for the 12-fold symmetry photonic quasicrystal lattices. The calculated transmission spectra of the PQC with incident angles along with 0, 5°, 10°, 15°, 20°, and 25° as indicated in Fig. 5a are presented in Fig. 5b. Due to the symmetry of this PQC lattices, the spectra would repeat for every 30° incident angle. The high transmission value in the spectra (blue color) indicates that the incident signal coupled into the PQC lattice resonant modes which are the band diagram areas. The low transmission (yellow color) regions indicate several photonic band gaps (PBGs) of the PQC structure. The ratio of high-to-low transmission is more than four order which show the PQC lattices take the strong effect to select the propagation modes in the device. The observed lasing actions occur around the band-edges of the PQC bandstructure, which are the boundaries between the high-transmission and low-transmission regimes in Fig. 5b. The flat dispersion curve near the band-edge implies a low group velocity of light and strong localization and lead to the lasing actions of the devices. These PBGs matched the emission wavelength of In_*x*_Ga_1−*x*_N/GaN with the corresponded normalized frequency are a/λ ≈ 0.88, 1.0, and 1.25 which were labeled as mode M_1_, M_2_, and M_3_. With the coupling between the PQC band-edge resonances and the emission from the InGaN/GaN layers, the emission efficiency and the light extraction at the specific wavelength would be further improved. The lasing action from GaN coupled to the high-frequency M_3_ could be achieved under sufficient excitation as our previous demonstration [43, 45]. For the regrown In_0.1_Ga_0.9_N and In_0.3_Ga_0.7_N which coupled to M_2_ and M_1_, the emission blue and green light would be boosted. Therefore, leveraging the coupling between the optical modes of PQC structure and In_*x*_Ga_1−*x*_N/GaN, efficient multicolor LEDs, LDs could be realized in such hybrid platform. The length of the nanorods in photonic crystal lattices is also important to generate the high-quality color enhancement. In this study, in order to achieve high-quality color enhancement, the photonic crystal nanorod length was etched to 1000 nm which is more than four times of the effective wavelength. To realize the multicolor emission from a single PQC device in the future, the multiple regrowth procedures should be added in the epitaxial process.

## Conclusions

In summary, a 12-fold symmetric GaN PQC nanopillars was fabricated using the NIL technology. High-efficiency blue and green color emissions from In_*x*_Ga_1−*x*_N/GaN MQWs were achieved with the regrowth procedure of the top In_*x*_Ga_1−*x*_N/GaN MQWs grown on these facets, with an In composition ratio: In_*x*_Ga_1−*x*_N/GaN-dependent InN fraction variations. The emission peaks were observed around 366-, 460-, and 520-nm wavelength resulting from In_0.1_Ga_0.9_N/GaN MQWs and In_0.3_Ga_0.7_N/GaN MQWs, respectively. These emission modes correspond to the band-edge resonant modes of the GaN PQC structure with FEM simulation. The methods of fabrication demonstrated a great potential to be a low-cost technique for fabricating semipolar {10-11} In_*x*_Ga_1−*x*_N/GaN LED to use in manufacturing multicolor light sources. We believe that GaN-based photonic quasicrystal lasers could be integrated into multicolor light source systems in the future.

## Data Availability

All data supporting the conclusions of this article are included in the article.
